# Family Reading

**Published:** 1871-03

**Authors:** 


### FAMILY READING.

THAT increasing intelligence and morality are lengthening the average duration of human life, cannot be disputed; it was twenty years two centuries ago; it is now over forty in civilized societies. It is just as true that increasing intelligence and a decrease in the moral or religious element will rapidly abridge the life of individuals, communities, and nations. Intelligence insures prosperity, pecuniary thrift; but unless there is a correspondent increase of the religious sentiment, of high moral principle, imposing proper restraints on the facilities of indulgences which wealth offers, individuals will die, communities will decrease, and nations will become extinct, from their very highest points of attained grandeur, of cultivation, of art and grace, of power and domain, and hoards of wealth. Egypt, and Babylon, and Greece, and Rome, pagan in principle and practice, went rapidly down to the deepest depths of human depravity and human degradation.

Leaving it to the pulpit to take care of the moral culture, occasion is here taken to suggest that it is of growing importance that every family claiming in the least to be cultivated, should not only take one good religious weekly publication and a respectable newspaper, but also some scientific journal which in its teachings will certainly elevate and refine, because it addresses the reflective faculties, and whatever makes a man think raises him upwards to a higher nature. Whatever makes the youngest child think, whatever tends to cultivate " thinking for themselves " in any household, goes far, very far indeed, to make of that household, within a very few years, men and women of which any community may well be proud.

*The Scientific American* is the first, the best, and the ablest of its kind in the world. Always of a high moral tone, elegant in its mechanical execution and getting up, it abounds, always abounds in practical matter of great value in the workshop, on the farm, in the dairy, the kitchen, the garden, and the orchard; it gives the earliest and most reliable information about all inventions and improvements, saving labor, economizing means, and adding immeasurably to household convenience and comfort. The very sight of it on a gentleman's centre table is a guarantee that there is intelligence in that household. Or suppose a father orders for his family *The Boston Journal of Chemistry*, coming out monthly, at a dollar a year; a daughter of eighteen, or the young wife, or the newly married mechanic or farmer, might imagine and will take it for granted, at least at first thought, that "there is nothing in it for me," and straightway begins to think of carbon, and oxygen, and hydrogen, and air, and a great variety of glassy and tin things half-broken, and bent, and battered out of their original propriety; but look for a moment at a part, a small part, of the contents of one single number.

Ought Water to be put in a Pan on a Stove or Furnace? How to do for Sick People. Collodion for protecting Silverware. A New Whitewash for Walls. To clean Paint. Management of Hyacinth and Tulip Bulbs. Buds and Blossoms out of Season. Use of Old Shoes. For Ringworm. Treatment of Dandruff. Varnish in Burns. To arrest Decay of Teeth. Perhaps there is not one in ten of all our readers who will not feel that it was worth a "quarter" to have that number, and would give it willingly to see what is said upon one single subject, upon which some particular person wants information. All have teeth, for example; all have dandruff; who would not be benefited by wanting to know "how to do for sick people?" And yet this is an old number for December, 1869, laid aside then for the happy occasion when there would be leisure to read it; and to-day only gives time to glance at the list of contents. Ah, what a terrible, what an everlasting "push" a city life is! Why, there is more useful, practical, and reliable information in this little dingy, smoked old number of the *Boston Journal of Chemistry* for six cents than in a whole year's reading of story newspapers, and novel-crammed monthlies at three and four dollars a year.

Of a kindred nature, larger and more valuable in a medicinal point of view, is *The Druggist's Circular and Chemical Gazette* of New York City, for family reading. Also *The Journal of Applied Chemistry*, for New York; *The Technologist*, especially adapted to engineering, manufacturing, and building, with a very large amount of other reading, calculated to make thinkers in every family, and to elevate any habitual reader of its valuable pages. Two dollars a year. Address American News Company, 121 Nassau Street, New York. These are but a few of the scientific publications adapted for family reading, improvement, and elevation, which happened to be at the moment on the Editor's table. And when we take into account the tons of trashy matter which overburden our railway trains every week in the shape of stories, novels, demoralizing narrations, and dauby engravings of all that is indelicate, degrading, and horrible, familiarizing the tender minds of the young with deeds of gougings, fightings, and murder, the subject of Family Reading merits the thoughtful consideration of every intelligent parent.

